# Genetic modulation of the *HTR2A* gene reduces anxiety-related behavior in mice

**DOI:** 10.1093/pnasnexus/pgad170

**Published:** 2023-06-20

**Authors:** Troy T Rohn, Dean Radin, Tracy Brandmeyer, Barry J Linder, Emile Andriambeloson, Stéphanie Wagner, James Kehler, Ana Vasileva, Huaien Wang, John L Mee, James H Fallon

**Affiliations:** Department of Biological Sciences, Boise State University, Boise, ID 83725, USA; Cognigenics, Eagle, ID 83616, USA; Cognigenics, Eagle, ID 83616, USA; Cognigenics, Eagle, ID 83616, USA; Cognigenics, Eagle, ID 83616, USA; Neurofit, Illkirch-Graffenstaden 67400, France; Neurofit, Illkirch-Graffenstaden 67400, France; Mirimus Inc., Brooklynn, NY 11226, USA; Mirimus Inc., Brooklynn, NY 11226, USA; Mirimus Inc., Brooklynn, NY 11226, USA; Cognigenics, Eagle, ID 83616, USA; Cognigenics, Eagle, ID 83616, USA; Department of Psychiatry and Human Behavior, University of California, Irvine, CA 92697, USA

**Keywords:** CRISPR, HTR2A, 5HT-2A receptor, anxiety, intranasal delivery

## Abstract

The expanding field of precision gene editing using CRISPR/Cas9 has demonstrated its potential as a transformative technology in the treatment of various diseases. However, whether this genome-editing tool could be used to modify neural circuits in the central nervous system (CNS), which are implicated in complex behavioral traits, remains uncertain. In this study, we demonstrate the feasibility of noninvasive, intranasal delivery of adeno-associated virus serotype 9 (AAV9) vectors containing CRISPR/Cas9 cargo within the CNS resulting in modification of the *HTR2A* receptor gene. In vitro, exposure to primary mouse cortical neurons to AAV9 vectors targeting the *HT2RA* gene led to a concentration-dependent decrease in spontaneous electrical activity following multielectrode array (MEA) analysis. In vivo, at 5 weeks postintranasal delivery in mice, analysis of brain samples revealed single base pair deletions and nonsense mutations, leading to an 8.46-fold reduction in mRNA expression and a corresponding 68% decrease in the 5HT-2A receptor staining. Our findings also demonstrate a significant decrease in anxiety-like behavior in treated mice. This study constitutes the first successful demonstration of a noninvasive CRISPR/Cas9 delivery platform, capable of bypassing the blood–brain barrier and enabling modulation of neuronal 5HT-2A receptor pathways. The results of this study targeting the *HTR2A* gene provide a foundation for the development of innovative therapeutic strategies for a broad range of neurological disorders, including anxiety, depression, attentional deficits, and cognitive dysfunction.

**Significance Statement**

Current therapies for anxiety and depression rely heavily on selective serotonin reuptake inhibitors that may impact numerous serotonergic receptor pathways, are fraught with side effects, and require daily dosing. Herein, we employed precision gene targeting to selectively knockdown the 5HT-2A receptor, one of the 15 serotonin receptor subtypes known to play a key role in anxiety and depression. To accomplish this goal, we used a noninvasive, intranasal delivery platform consisting of two adeno-associated virus (AAV) vectors containing plasmids for CRISPR/Cas9 and guide RNA, respectively, to knockdown the 5HT-2A receptors in anatomical and functional connectomes implicated in both anxiety and depression. Our results indicated administered AAV–CRISPR–Cas9 significantly reduced anxiety, thus demonstrating that complex behavioral traits can be directly modified long-term through genetic editing.

## Introduction

CRISPR is a powerful tool that has the potential to revolutionize genomic engineering by potentially treating various diseases (1). The CRISPR/Cas9 system consists of two parts: the Cas9 protein which is the nuclease capable of inducing double-strand DNA breaks and the guide RNA (gRNA) which is designed to target any specific DNA sequence in the genome. Once double-strand breaks are generated, the DNA is repaired by either nonhomologous end joining (NHEJ) or homologous-directed repair (HDR) pathways that induce insertions or deletions (indels) at the target site (2). Dozens of human clinical trials are currently underway using the CRISPR/Cas system, including those for sickle cell anemia and several forms of cancer (3). Despite the medical promise of CRISPR, the use of this system in treating central nervous system (CNS) disorders is challenged by the noninvasive delivery across the blood–brain barrier (BBB). It has been estimated that more than 98% of small-molecule drugs and nearly 100% of large-molecule drugs, such as the CRISPR/Cas9 system, are precluded from drug delivery to the brain as a result of the BBB (4). Efforts to overcome this hurdle include the use of adeno-associated viral (AAV) vectors, such as AAV9, due to their ability to deliver stable, long-lasting transgene expression in nondividing cells (5). However, even this platform has been met with limited success in penetrating the BBB (6).

In the present study, we investigated whether intranasal delivery of CRISPR/Cas9 could bypass the BBB and lead to knockout of the *HTR2A* gene in neuronal populations (*PCT/US2022/050947I*). The 5HT-2A receptor is implicated in both anxiety and depression disorders (7–10), and current legacy treatments with selective serotonin reuptake inhibitor (SSRI) compounds are fraught with side effects, show low efficacy, and require daily dosing (11, 12). Therefore, there is a pressing need for newer, more effective therapeutic agents to treat anxiety and depression. Results from the current study demonstrate that AAV–CRISPR/Cas9 intranasal delivery in mice leads to CNS infiltration, neuronal modulation of the 5HT-2A receptor, and reduction in anxiety. These results highlight a new delivery platform to provide CRISPR/Cas9 into the brain and proof of concept that certain behavioral traits can be directly modified long-term. Noninvasive CNS delivery of CRISPR/Cas9 may have important implications for the next generation of psychotropic medications, particularly for patients exhibiting treatment-resistant anxiety or depression (13, 14).

## Methods

### gRNA design

**Fig. 1.**
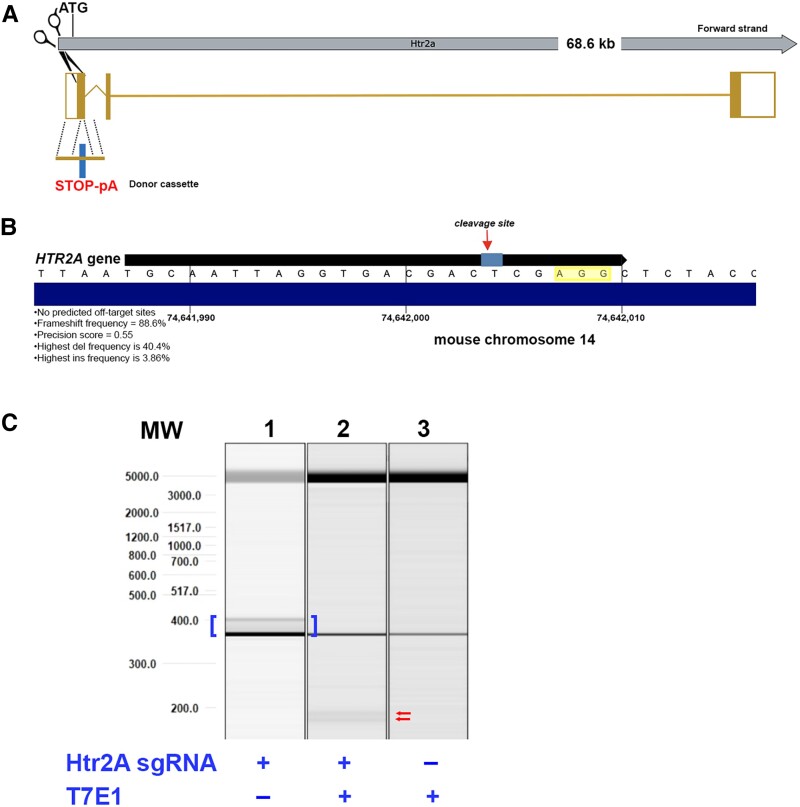
Targeting strategy to knock out the mouse *HTR2A* gene using CRISPR/Cas9. A) The *HTR2A* mouse gene encodes a single protein-coding transcript, *Htr2a-201*, located on chromosome 14. Two in silico strategies were designed to knock out the gene with both strategies utilizing the same cut site for Cas9 in the first coding exon. In the first strategy, Cas9-directed double-strand break near the start site (ATG) would be repaired by NHEJ, introducing random indels and potential frameshift mutations. The second strategy CRISPR/Cas9 would cut in the first coding exon and HDR to insert a STOP-pA cassette to terminate transcription of the *HTR2A* gene. For details on this donor sequence, see the Methods section. B) gRNA design is depicted in black with the predicted Cas9 cleavage site between the T and C (arrow) and the PAM sequence, AGG. This gRNA predicts to have no off-target sites, a frameshift frequency of 88.6%, and a precision score of 0.55. C) Cleavage efficiency assessment in mouse ESCs. Naïve mouse ESCs were transfected with gRNA and Cas9, and transfected cells were selected by puromycin selection. Cleavage efficiency was determined by digesting annealed PCR products with T7E1, and fragments were analyzed following gel electrophoresis. Brackets in lane 1 denote PCR amplicon of the predicted size (375 bp) along with editing products. Arrows denote expected sizes post T7E1 cleavage (196 and 179 bp, respectively).

The *HTR2A* mouse gene encodes a single protein-coding transcript, Htr2A-201, located on chromosome 14. Two in silico strategies were designed to knock out the gene with both strategies utilizing the same cut site for Cas9 in the first coding exon (Fig. 1A). In the first strategy, a Cas9-directed double-strand break near the start site (ATG) would be repaired by NHEJ, introducing random indels and potential frameshift mutations. In the second strategy, CRISPR/Cas9 would cut in the first coding exon and HDR systems would lead to insertion of a STOP-pA cassette to terminate transcription of the *HTR2A* gene. The gRNA chosen in both cases, TGCAATTAGGTGACGACTCGAGG (*PCT/US2022/050947*), would give no predicted off-target cut sites, produce an 86.6% frameshift frequency, and a precision score of 0.55. Figure 1B depicts the cleavage site of spCas9 three nucleotides upstream of a consensus PAM sequence, AGG (highlighted yellow, Fig. 1B). An additional 200 bp knockin STOP-pA donor cassette was manufactured that would insert two stop codons shown in bold below:

GAAGACAATATCTCCCTGAGCTCAATTCCAAACTCCTTAATGCAATTAGGTGAC**tAgtga**ATTCAACTTGTTTATTGCAGCTTATAATGGTTACAAATAAAGCAATAGCATCACAAATTTCACAAATAAAGCATTTTTTTCACTGCTCGAGGCTCTACCCTAATGACTTCAACTCCAGGGATGCTAACACTTCCGAAGCC

### T7 endonuclease 1 detection assay

To evaluate the activity of spCas9 using the above gRNA design, T7 endonuclease 1 (T7E1) assays were carried out. This assay detects heteroduplex DNA that results from annealing DNA strands that have been modified after a gRNA/Cas9-mediated cut to DNA strands (15). Briefly, following preparation of genomic DNA, amplification of the target site by PCR was carried out using designed primers on either side of the target sequence: Htr2A LF1: TGACTCGCTAGTCTCTCCACAC and HTr2A LR1: GCTTCCCATGGAGGGTGG. Following amplification, T7E1 digests were performed to detect potential mismatches at the target site. In silico design and in vitro validation of gRNA as well as spCas9 cutting efficiency was performed by Mirimus Inc. (Brooklyn, NY).

### Validation of gRNA in mouse embryonic stem cells and generation of F0 founder knockout mice for the HTR2A gene

To test in vitro and in vivo knockout strategy, experiments were performed in mouse wild-type embryonic stem cells (ESCs). ESCs were transfected with spCas9, donor sequence containing premature stop codons, and gRNA. Targeted knockout of the 5HT-2A receptor mouse gene in ESC cells was performed by Mirimus Inc. (Brooklyn, NY). Injection of modified ESC cells and generation of founder knockout mice was conducted by Charles River (Wilmington, MA).

### AAV vector design

**Fig. 2.**
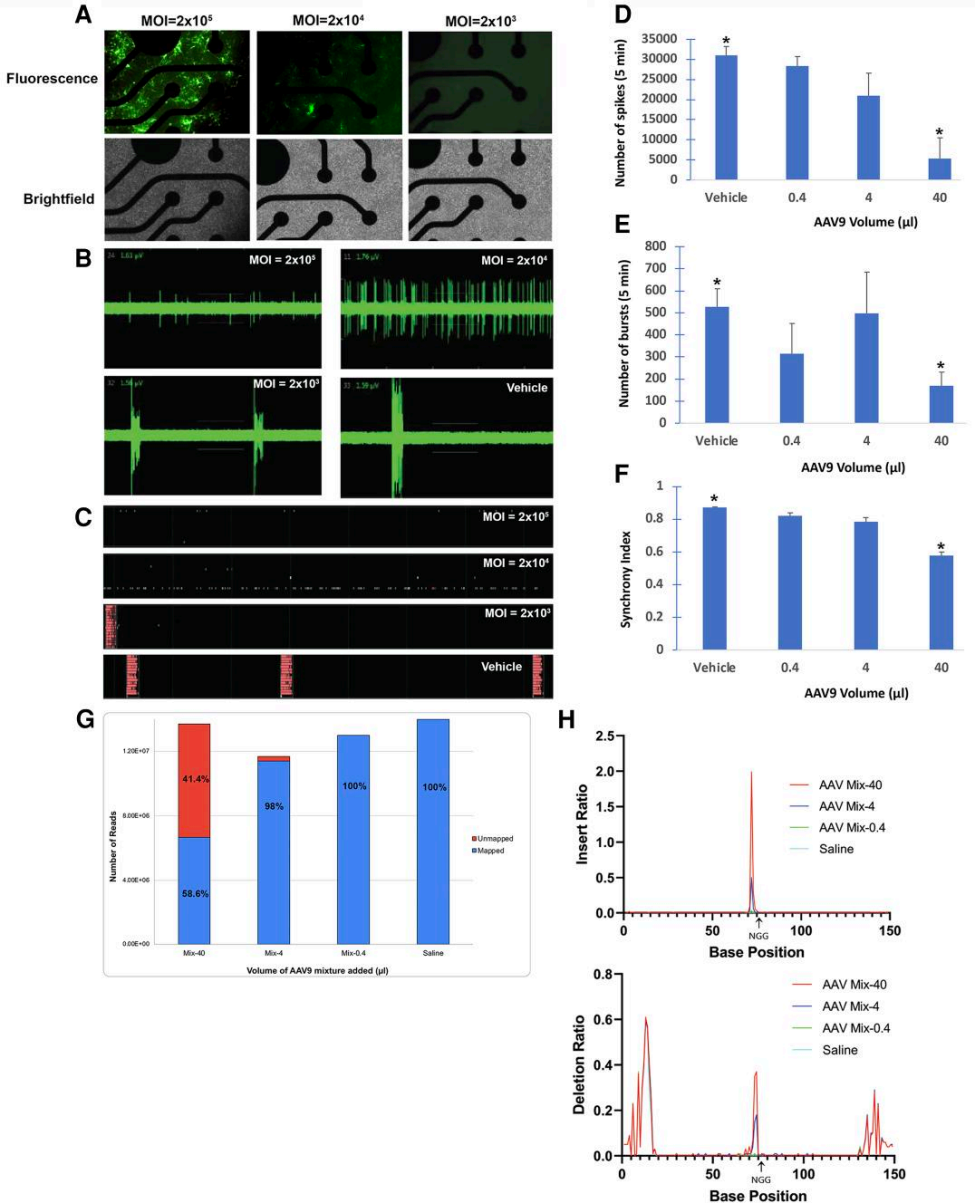
In vitro exposure of AAV copackaged *Cas9* DNA with *HTR2A*-targeting gRNA leads to a decrease in spontaneous electrical activity of primary mouse cortical neurons. MEA analysis was performed in mouse cortical neurons following treatment with a mixture of AAV9 vectors containing AAV9–Mecp2–spCas9–sPA and AAV9–GFP–U6–mHtr2a–gRNA–ssODN at various concentrations indicated by the MOI. Neurons were treated at day 6, and MEA analyses were performed on day 14 (see the Methods section for details). A) Transduction was confirmed in primary cortical neurons by detecting GFP fluorescence. As shown, strong GFP fluorescence was observed at a MOI of 2 × 10^5^, with the intensity decreasing significantly at 2 × 10^4^, and 2 × 10^3^ MOI, respectively. B) There are 16 electrodes in each well, and each panel represents the recording of the real-time signal of one selected electrode at different MOIs. The gray threshold lines represent the voltage of the baseline noise. If an electrode signal is beyond the threshold, it will be recorded as a spike. As the MOI increased, the number of spikes decreased significantly as compared with vehicle. C) Shown are continuous spike signals of all 16 electrodes within 50 s. Each row presents an electrode. The bursts are shown as vertical bars. A network of bursts is a coordinated cluster of spiking across multiple electrodes. There were network bursts in the vehicle and lowest MOI, but not at higher MOIs including 2 × 10^4^ and 2 × 10^5^. D–F) Quantification of MEA analysis showing the total number of spikes over the duration of the analysis D), the number of network bursts defined as a cluster of spikes across all electrodes E), and the synchrony index, which indicates a unitless measure of synchrony between 0 and 1. Values closer to 1 indicate higher synchrony. In all cases, exposure of neurons to 2 × 10^5^ MOI led to a significant decrease in the number of spikes (83% decrease compared with vehicle controls, *P* = 0.0007, *N* = 3) D); in the number of bursts (68% decrease compared with vehicle controls, *P* = 0.014, *N* = 3) E); and a decrease in the synchrony index (34% decrease compared with vehicle controls, *P* = 0.0002, *N* = 3) F). G) and H) are identical in terms of experimental procedure as A–F), with the exception that genomic DNA was collected following treatments and next-generation targeted sequencing was performed as described in the Methods section. G) depicts the efficiency analysis of gene editing in a stacked column graph with the total number of reads (unmapped versus mapped) for each treatment group. The unmapped reads represent the counts of sequence that were inconsistent with the reference sequence (*HTR2A* gene). The mapped reads represent the counts of sequence that were consistent with the reference sequence. At the highest concentration, MOI = 2 × 10^5^, a 41.4% gene editing efficiency was observed, followed by a 2% at MOI of 2 × 10^4^. In H), indels within the target sequence of the HTR2A gene (149 bp) are shown with the top panel representing the insertion ratio, while the bottom pattern representing the deletion ratio. In both cases, the highest MOI concentration led to a sharp increase in indel formation within the target site. NGG in both graphs indicates the PAM sequence. AAV9 volumes of 40, 4, and 0.4 µL correspond to a MOI of 2 × 10^5^, 2 × 10^4^, and 2 × 10^3^, respectively (see Table 1 of main text).

Two different adeno-associated virus serotype 9 (AAV9) vectors were designed to deliver spCas9 and gRNA to CNS neurons. The design of two vectors was necessarily based on the limited carrying capacity of 4.7 kb for AA9 viruses (16), thus we created dual AAV9 systems to expand the capacity. The first AAV9 expressed spCas9 under a neuronal-specific promoter, MeCP2 (∼4.2 kb) (Fig. 2A), and the spCas9 vector utilized the PX551 plasmid from Addgene (pAAV-pMecp2-SpCas9-spA) (17). The second AAV9 vector consisted of the gRNA sequence, 200-bp SS ODN donor template, and a green fluorescent protein (GFP) reporter under the U6 promoter (AAV-GFP-ssODN-U6-gRNA). Large-scale transfection of plasmids was carried out in HEK293 cells and purified through a series of CsCl centrifugations. Titer load (in genome copy number per mL, or GC/mL) was determined through quantitative real-time PCR, with typical yields giving 2.0 × 10^13^ GC/mL. Both AAV9 vectors were stored in PBS with 5% glycerol at −80°C until used. Design, manufacturing, and purification of AAV9 vectors used in this study were performed by Vector Biolabs (Malvern, PA).

### Primary mouse cortical neuron cultures and multielectrode array analysis

The materials MEM, PS, GlutaMAX, FBS, Neurobasal, and B27 were from Gibco. The microscope used was an Evos XL Core. Twenty-four-well multielectrode array (MEA) plates were coated with 500 µL 0.07% PEI and incubated for 1 h. Plates were then washed four times in sterile deionized water and dried overnight in a biosafety cabinet. Primary cortical neurons from fresh mouse brain embryos were isolated and plated onto coated 24-well plates at a density of 5 × 10^5^ cells/well. Cortical neurons were maintained in Neurobasal-A Medium, supplemented with B27, GlutaMAX, and antibiotics (100 U/MI penicillin and 100 µg/mL streptomycin). Cultured neurons were incubated at 37°C and 5% CO_2_ and half the media was exchanged with fresh, complete media every 3 days. The AAV-treated cocktail consisted of an equal mixture of AAV9–gRNA–U6–GFP and AAV9–Mecp2–spCas9 suspended in 0.9% NaCl (saline). The final concentration of each AAV9 vector in this mixture was 2.5 × 10^12^ GC/mL. Infection of cortical neurons was based on the multiplicity of infection (MOI), which refers to the number of viral particles per cell. Three different MOI concentrations were used: 2 × 10^5^ (which is equivalent to a total number of viral particles of 1 × 10^11^), 2 × 10^4^, and 2 × 10^3^ (Table 1). Control neurons received vehicle (40 µL of saline) equivalent to the volume of fluid given at each MOI. Treatment was done in triplicate. Treatment occurred on day 6 for 24 h at 37°C at which time the media was replaced with fresh, complete media. MEA analysis was then performed on day 14. Primary mouse cortical neuron cultures and MEA analysis were performed by Creative Biolabs (Shirley, NY).

**Table 1.** Experimental setup for treating primary mouse cortical neurons with CRISPR/Cas9 cargo, in vitro.

Group	AAV9 mixture (µL)	Saline (µL)	MOI
A	40	—	2 × 10^5^
B	4	36	2 × 10^4^
C	0.4	39.6	2 × 10^3^
D	—	40	0

### Animals for light–dark and marble burying tests

All animal care and experimental procedures were performed in accordance with institutional guidelines and were conducted in compliance with French Animal Health Regulation, at Neurofit SAS (Illkirch, France). Male CD-1 mice, 4–5 weeks old, were obtained from Janvier (Le Genest St Isle, France), and group-housed five or six per cage in a temperature-controlled room (21–22°C) with a light–dark cycle (12 h/12 h; lights on: 5:30 PM–5:30 AM; lights off: 5:30 AM–5:30 PM) with food and water available ad libitum. Mice were given at least 1-week acclimatation to the animal facility before starting any experimental procedures.

### Test formulation and intranasal administration

The AAV-treated group consisted of an equal mixture of AAV9–gRNA–U6–GFP and AAV9–Mecp2–spCas9 suspended in 0.9% NaCl (saline). The concentration of AAV9 stock mixture was ∼5.0 × 10^12^ GC/mL for mice tested in the light–dark and marble burying test, respectively. For each behavioral test, a new, independent batch of AAV9 vectors was synthesized and prepared accordingly. The AAV cocktail was administered twice on day 1 (morning and afternoon) 5 weeks before the behavioral tests. For intranasal delivery of AVV, mice were hand-restrained with the nose positioned to facilitate the dosing. A meniscus of AAV solution droplet (10 µL per nostril) was then formed at the tip of the micropipette and presented for inhalation in each of the nares of the mouse. Each mouse received a total of 40 µL of AAVs equivalent to ∼2 × 10^11^ viral particles (vehicle-treated animals received 40 µL of saline for each treatment following the same protocol).

### Light–dark behavioral test

In this task, mice were given a choice between exploring a brightly lit chamber or a dark chamber; anxious mice will spend more time in the dark chamber. The apparatus consisted of two polyvinyl chloride (PVC) boxes (19 × 19 × 15 cm) covered with Plexiglas. One of these boxes was opaque and not illuminated. The other box was illuminated by desk lamp placed above and providing ∼2,000 lx. An opaque plastic tunnel (5 × 7 × 10 cm) separated the dark box from the illuminated one. A camera linked to a video tracking system (Viewpoint, France) was used to monitor the behavior of the mouse in the lit box. Animals were placed individually in the lit box, with their head directed towards the tunnel. The time spent in the lit box and the number of transitions between the two boxes was recorded over a 5-min period after the first entry of the animal in the dark box. The total distance traveled in the lit box was also recorded. The apparatus was cleaned between each animal using 70% alcohol.

### Marble burying test

The apparatus consisted of transparent polycarbonate cages (30 cm × 18 cm × 19 cm) containing a 5-cm layer of fine sawdust bedding and 20 opaque marbles (diameter: 1.5 cm) spaced evenly along the walls of the cage. Each animal was placed individually in the cage where it remained for a 20-min test session. On termination of the test session, the animals were removed from the cage and the number of marbles with at least two-thirds of the marbles buried in the sawdust was counted. Anxious mice will bury more marbles.

### Animals for elevated plus maze test

Male C57Bl/6J mice from Jackson Labs were utilized. Mice were received at 6–7 weeks old and allowed to acclimate for 1–2 weeks prior to testing at an average age of 8 weeks. All animals were examined, handled, and weighed prior to initiation of the study to assure adequate health and suitability. During the course of the study, 12/12 light/dark cycles were maintained. Chow and water were provided ad libitum for the duration of the study. Each mouse was randomly assigned across the treatment groups. The test was performed during the animal's light cycle phase.

### Elevated plus maze

The elevated plus maze (EPM) has two opposite open arms and two closed arms. Mice will sometimes explore and spend time in the open arms, but anxious mice tend to first enter and spend most of the test time in the closed arms. The maze (Hamilton Kinder) consists of two closed arms (14.5 *h* × 5 *w* × 35 cm length) and two open arms (6 *w* × 35 *l* cm) forming a cross, with a square center platform (6 × 6 cm). All visible surfaces are made of black acrylic. Each arm of the maze is placed on a support column 56 cm above the floor. Antistatic black vinyl curtains (7′ tall) surround the EPM to make a 5′×5″ enclosure. Animals are brought into the experimental room at least 1 h before the test to acclimate. Mice were placed in the center of the EPM facing the closed arm for a 5-min run. All animals were tested once. The number of entries in the closed and open arms was automatically recorded by a computer, and the EPM was thoroughly cleaned after each mouse. Testing was conducted at week 5 of intranasal dosing as described above, with each mouse (*n* = 15) receiving a total of 40 µL of AAVs equivalent to ∼2 × 10^11^ viral particles. Vehicle-treated animals (*n* = 15) received 40 µL of saline for each treatment following the same protocol. Treatment and EPM behavioral analyses were performed by PsychoGenics (Paramus, NJ). The behavioral tests were conducted according to established protocols approved by the PsychoGenic's IACUC committee and their standard operation procedures (SOP).

### Tail suspension test

Mice will tend to actively struggle to right themselves when suspended upside down by the tail. The tail suspension test can be an indicator of depression and measures the amount of time the mouse struggles to right itself before “giving up” and becoming immobile. A short time to immobility indicates depression. Mice are acclimated to the test room at least 1 h prior to the commencing the test. Each animal is suspended into the tail suspension chamber (white PVC cubicles measuring 33 × 33 × 31.75 cm; Med Associates, Inc., St. Albans, VT) by a piece of transparent (Scotch) tape attached to the tail, from about the midtail, with ∼2 cm of tape past the end of the tail. The observation period begins immediately after the animal is suspended and runs for a maximum of 10 min. Data are automatically collected in 1-min bins. Measurements of immobile time are acquired by the tail suspension software. Data are presented as the total immobile time over the test duration. Treatment and behavioral analyses were performed by PsychoGenics (Paramus, NJ). The behavioral tests were conducted according to established protocols approved by the PsychoGenic's IACUC committee and their SOP.

For each behavioral trial described above, a new lot of AAV9 vectors was synthesized and used for intranasal delivery.

### Tissue preparation

Immediately following behavioral analysis, mice were anesthetized with 5% isoflurane/oxygen mixture and sacrificed by decapitation. Brains with olfactory bulbs were extracted and fixed in 4% formalin for 48 h and transferred to vials containing 1% formalin in PBS buffer. Brain samples were stored at 4°C. Some brains were flash frozen and stored at −80°C prior to RT-PCR or next-generation sequencing (NGS) analysis.

### Statistical analysis

Statistical analyses of all behavioral, ImageJ, and quantitative qPCR results were assessed by one-tailed independent Student's *t* test unless otherwise specified.

### Quantitative real-time qPCR

Total RNA was extracted from frozen brains using a standard extraction protocol with TRIzol, dissolved in diethylpyrocarbonate-treated deionized water, and quantified. Following reverse transcription, qPCR was carried out using the following primers: Primer-F: 5′-AGAGGAGCCACACAGGTCTC-3′ and Primer-R: 5′-ACGACAGTTGTCAATAAAGCAG-3′. The relative expression was determined by calculating the 2^−ΔCt^ value. The 2^−ΔΔCt^ value was calculated and normalized to calculate a fold difference between the two vehicle controls and AAV9-treated groups. The RNA extraction and qPCR were performed by Creative Biogene (Shirley, NY, USA).

### Targeted region sequencing

To identify potential CRISPR/Cas9 mutations in primary mouse cortical neurons and treated mouse brain samples, NGS was performed of PCR targeted amplicons as previously described (18). Briefly, following genomic DNA isolation, PCR was used with site-specific primers to amplify the target region. Primers used were the following [length: 149 bp; sgRNA: 57–79 bp (italicized); PAM: 77–79 bp (in bold)]:

Htr2a-TRS-F1: 5′-CATGGAAATTCTCTGTGAAGAC-3′
Htr2a-TRS-R1: 5′-CAGCATCAATTGTCCAGTTCG-3′
Htr2a-TRS-R1: 5′-CAGCATCAATTGTCCAGTTCG-3′
CATGGAAATTCTCTGTGAAGACAATATCTCCCTGAGCTCAATTCCAAACTCCTTAA*TGCAATTAGGTGACGACTCG**AGG***CTCTACCCTAATGACTTCAACTCCAGGGATGCTAACACTTCCGAAGCCTCGAACTGGACAATTGATGCTG

Following amplification, PCR for NGS adaptors was performed followed by NGS and data analysis. Genomic DNA isolation and targeted region sequencing were performed by Creative Biogene (Shirley, NY, USA).

### Fluorescence microscopy

Following dehydration, 4-µm paraffin-embedded, sagittal sections were cut lateral to the midline and used for immunofluorescence labeling. Briefly, all tissue sections were labeled with anti-GFP antibody (rabbit mAB #2956) 1:1,000 (Cell Signaling Technology, Inc., Danvers, MA, USA) or anti-5HT-2A receptor antibody (rabbit polyclonal, #24288) at 1:500 dilution (Immunostar, Hudson, WI). Secondary antibodies were conjugated to FITC or Cy3. DAPI was used as a nuclear stain. Whole slide scanning was performed using a Pannoramic Midi II scanner using a 40× objective lens with optical magnification of 98×, 0.1 µm/pixel. All sectioning, immunolabeling, and capturing of images were performed by iHisto (Salem, MA).

### ImageJ quantification

The level of 5HT-2A receptor fluorescence was quantified using ImageJ software (19). This was accomplished by capturing 50× immunofluorescence images from three different fields (cortex, cerebellum, and subcortical regions) in three separate tissue sections from each group (vehicle control or AAV-treated). All data were expressed as the mean gray value ± SEM. The mean gray value reflects the sum of the gray values of all the pixels in the selected area divided by the number of pixels. The area of selection in square pixels was not significantly different between vehicle controls (676,181) and AAV-treated (671,647), *P* = 0.639, *N* = 9 for each group.

## Results and discussion

### In silico gRNA design and validation

Two different strategies involving either HDR or NHEJ repair systems were employed to knock down the mouse *HTR2A* gene using CRISPR/Cas9. Both strategies involved directing spCas9 to the first coding exon of the mouse *HTR2A* gene using the following gRNA sequence: TGCAATTAGGTGACGACTCGAGG (*PCT/US2022/050947*). One strategy employed the use of a single-strand, 200-bp knockin STOP-pA donor cassette (SS ODN template) that would insert two stop codons in the first coding exon (Fig. 1A). This system requires HDR, a process that mainly occurs in the S and G2 phases of the cell cycle and is thought to rarely take place in mature neurons (20). However, a recent study has demonstrated virus-mediated HDR gene editing by CRISPR/Cas9 in postmitotic neurons (21). In this study, the authors used a dual AAV9 system to demonstrate HDR-mediated genome editing with CRISPR/Cas9 in postmitotic neurons in the adult mouse brain (21). Therefore, we used a similar approach to test this as a possible gene editing pathway.

If HDR-mediated insertion of the SS ODN sequence does not occur, it is still possible to produce potential genomic changes in postmitotic neurons by random indels induced by Cas9 cleavage and non-NHEJ repair introducing premature stop codons (Fig. 1B). Thus, our strategy was to ensure potential gene editing within neurons regardless of whether HDR or NHEJ was the predominant repair systems.

Validation of Cas9 cleavage of this site using the above gRNA was confirmed in mouse ESC cells. Naïve mouse ESCs were transfected with gRNA and Cas9 and cells were selected by puromycin selection. Because the presence of a SS ODN template does not alter cleavage efficiency, this was not included. Cleavage efficiency was determined by digesting annealed PCR products with T7E1 and fragments were analyzed following gel electrophoresis. As shown in Fig. 1C, the gRNA targeted Cas9 to generate double-strand breaks, producing the expected size products of 196 and 179 bp following cleavage of the predicted 375 bp amplicon. The faint band in lane 1 may reflect many products that most likely are insertions, hence the longer size of the products (blue brackets, Fig. 1C). Overall, the sgRNA cleavage efficiency was low. However, because we were aiming for both NHEJ- and HDR-based editing possibilities, a balanced approach was taken; thus, efficiency is less relevant than the placement of the cleavage site with respect to the homology arms. In other words, HDR efficiency is not in a linear correlation to Cas9 cutting efficiency. Confirmation that insertion of the SS ODN stop template was accomplished following HDR repair was obtained in F0 founder mice following injection of gene-edited ESC cells into embryos and transferred to surrogate female mice. F0 offspring demonstrated the correct insertion of the SS ODN sequence containing the two premature stop codons (Figs. S1 and S2).

### Delivery platform of CRISPR/Cas9 utilizing AAVs

Following verification of the gRNA and Cas9 specificity, we designed a delivery platform to deliver these reagents in vivo. AAVs have largely been used to deliver CRISPR/Cas9 cargo for several reasons including their low immunogenicity, good safety profile, and long-term expression in nondividing cells (16). We chose the AAV9 serotype, which has been demonstrated to be a highly efficient vector for neurons in the CNS and robust transgene expression throughout the CNS (22, 23). It has been reported that AAV can serve as a donor template for HDR at higher efficiencies than nonviral approaches (24, 25). Moreover, the combined use of endonuclease to induce double-strand breaks with AAV has been reported to increase the frequency of AAV-mediated gene targeting by several orders of magnitude (25). One limitation of AAV vectors is a limited packaging capacity restricted to ∼4.7 kb, which necessitates the design of two different AAV9 vectors for CRISPR/Cas9 delivery (26). Therefore, we constructed two different AAV9 vectors, AAV9(1) and AAV9(2), with the first vector having the PX551 plasmid from Addgene (17), which allows for expression of spCas9 under the neuronal-specific promoter, MeCP2 (AAV9–Mecp2–spCas9–sPA). This promoter was chosen due to its small size (229 bp), thus allowing for packaging in the AAV9(1) vector. The second vector contained the gRNA sequence together with the knockin SS ODN under the U6 promoter (AAV9–GFP–U6–mHtr2a–gRNA–ssODN). This AAV9 vector also contained the GFP reporter gene to provide a visual proxy of gRNA expression. Typical viral titers were on the order of 2.0×10^13^ GC/mL.

### AAV exposure of primary mouse cortical neurons with Cas9 DNA and a HTR2A-targeting gRNA leads a decrease in spontaneous electrical activity

The serotonin 5HT-2A receptor is the major excitatory receptor subtype in the cortex; therefore, we examined whether exposure of primary culture neurons to our AAV9 cocktail would lead to a decrease in electrical activity as measured by MEA. In this case, we measured the spontaneous activity of networks following treatment by recording field potentials. The advantage of MEA is that it can generate high-throughput readout of neuronal populations with the placement of multiple electrodes recording at once rather than individually. We treated primary culture neurons at day 6 with a serial dilution of AAV9 vectors consisting of Cas9 plasmids and HTR2A–gRNA–ssODN at equal concentrations as described in the Methods section and performed MEA on day 14. As shown in Fig. 2, we observed a concentration-dependent effect on MEA parameters including a significant decrease in the total number of spikes (Fig. 2D), the number of bursts (Fig. 2E), and in the synchrony index (Fig. 2F). Importantly, there was no evidence of toxicity at any concentration following treatment, and neurons were healthy with no rupture of neurites. To confirm that AAV9 treatment led to gene editing, genomic DNA was isolated following treatment and next-generation targeted sequencing was performed as described in the Methods section. As shown in Fig. 2G, the ratio of unmapped reads with regard to mapped reads (the reference *HTR2A* gene) was Mix-40 >Mix-4 > Mix-0.4 = Saline. This can be interpreted to suggest that the highest concentration (MOI of 2 × 10^5^) resulted in the highest level of gene editing (41.4%). In Fig. 2H, the insertion/deletion ratio indicated a sharp increase at the target site in the Mix-40 and Mix-4 groups, with few mutations observed at the lowest AAV9 mixture as well as the control group. Taken together, these data support the conclusion that the AAV9–CRISPR/Cas9 mixture is nontoxic, induces gene editing, and decreases the collective electrical activity of cortical neuron networks in vitro.

### Intranasal delivery and validation of gRNA–GFP expression in the CNS

**Fig. 3.**
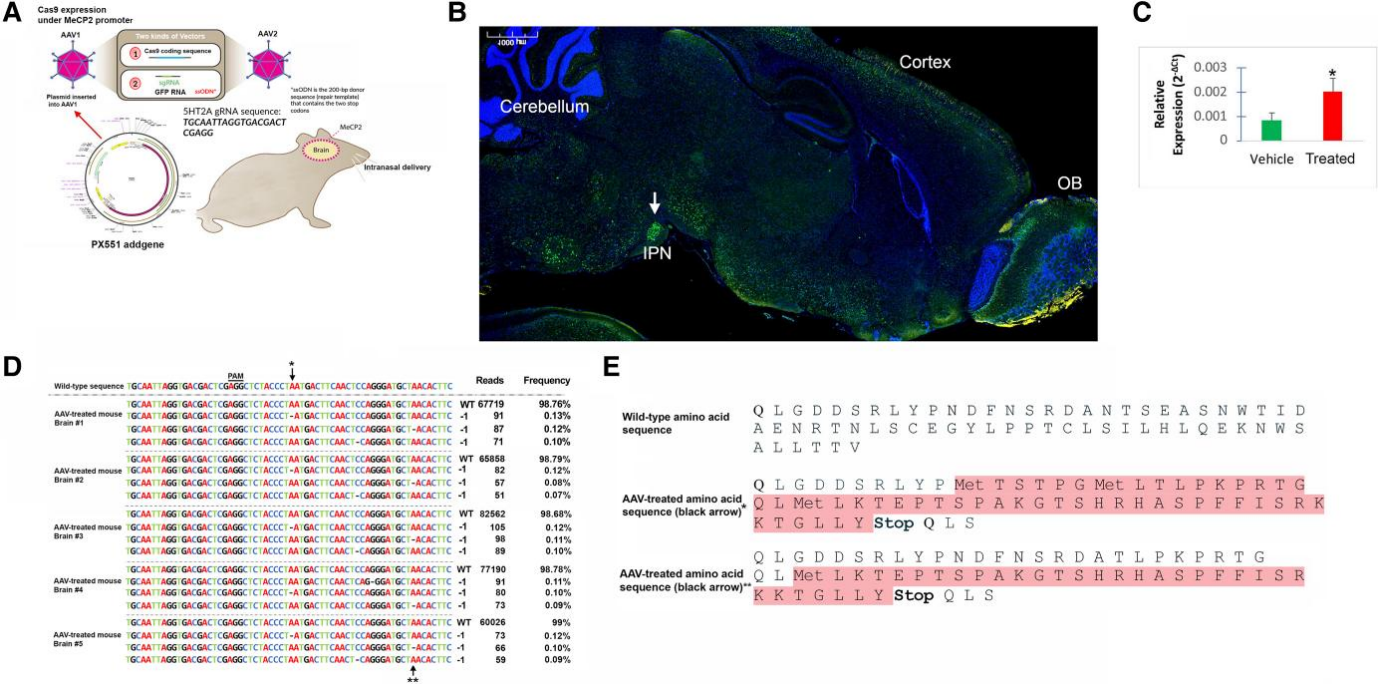
Intranasal delivery of AAV9 vectors containing Cas9 and gRNA receptor leads to down-regulation of 5HT-2A receptor mRNA. A) Experimental workflow (see Methods for details). AAV9–Mecp2–spCas9–sPA and AAV9–GFP–U6–mHtr2a–gRNA–ssODN were synthesized and typical viral titers were on the order of 2.0 × 10^13^ GC/mL. The plasmid design for expression of spCas9 under the neuronal-specific promoter, MeCP2, was from Addgene (17). Separately, AAV9 vectors were constructed that contained the gRNA for the *HTR2A* gene with a GFP coexpression system. Both vectors were mixed at equal concentrations and delivered intranasally via a micropipette tip (∼2.0 × 10^11^ viral particles). B) Representative, low-field immunofluorescence sagittal image following treatment, fixation in formalin, and immunolabeling using a specific antibody against GFP (1:1,000). GFP-positive neurons positively transfected with gRNA were identified in most brain regions including the olfactory bulb (OB), cortex, cerebellum, and numerous subcortical areas including the IPN, a major connectome for stress-mediated pathways. C) Data show the results of qPCR real-time assays to analyze mRNA levels of Htr2a following extraction of total brain RNA from frozen brain tissue in either vehicle controls (green bar) or CRISPR/Cas9 treated (bar labeled ‘treated’). Results display the *relative change* in expression. Real-time PCR results represent a total of *N* = 5 animals for each group performed in triplicate ± SEM. Asterisk denotes significant difference between the two groups, *P* ≤0.05. D) NGS analyses on-target effects in five individual mouse brain samples were analyzed and the most prevalent reads identified are shown. The profile mutations induced by *HTR2A*-targeting AAV-CRISPR/Cas9 revealed single base pair deletions indicated by the −1 symbol in adenine, which occurred in the exact positions for all five animals. E) The corresponding predicted amino acid sequences are presented for the two adenine indels with both mutations leading to a nonsense introduction of a premature stop codon. *Denotes single base pair deletion of adenine closest to the PAM sequnce. **Denotes single base pair deletion of adenine farthest from the PAM sequence. PAM, protospacer adjacent motif; WT, wild type.

For in vivo delivery in mice, we used the intranasal route (*PCT/US2022/050947*) because it bypasses the BBB and represents a practical, noninvasive method for delivery of CRISPR/Cas9 cargo. Intranasally administered therapeutics reach the CNS via the olfactory and trigeminal neural pathways that innervate the nasal cavity (27). Further delivery to the CNS occurs through the rostral migratory stream (RMS). The RMS is a specialized migratory route along which neuronal precursors that originate in the subventricular zone of the brain migrate to reach the olfactory bulb (28). Previous studies have demonstrated that intranasal administration of a fluorescent tracer allows agents to be distributed throughout the entire brain including the olfactory bulb, cortex, and cerebellum (29). Previous studies have supported the intranasal route as a viable strategy to deliver nerve growth factor gene inserts in human Alzheimer patients (30). In the present study, intranasal administration of an equal mixture of AAV9(1) and (2) vectors (final concentration of mixture was ∼2.0 × 10^11^ viral particles) resulted in widespread expression of GFP/gRNA throughout the CNS including the olfactory bulb, cortex, and subcortical areas including the interpeduncular nucleus (IPN) (Fig. 3), a major connectome for stress-mediated pathways (31). In all in vivo experiments, mice were treated intranasally on day 1, and immunofluorescent detection of GFP/gRNA was undertaken 5 weeks later. This concentration of AAV9 vectors and the time point chosen were based on a previous study examining neonatal intracerebroventricular injection of AAV9 (32).

### Intranasal AAV delivery of Cas9 DNA and a HTR2A-targeting gRNA leads to frameshift mutations and subsequent reduction in HT2RA expression

Next, we sought to confirm whether intranasal delivery of AAV9 vectors could lead to a decrease in the relative mRNA expression of the *HT2RA* gene. Following treatment of mice on day 1, mice were sacrificed 5 weeks post intranasal treatment and brains were snap-frozen. Total RNA was extracted from vehicle control (*N* = 5) or AAV-treated mice (*N* = 5) and real-time PCR was performed as described in the Methods section. Treatment led to a significant difference in the relative expression between the two groups (*P* = 0.05) (Fig. 3C). Normalized data indicated an 8.47-fold decrease in *HTR2A* expression. To analyze gene editing, whole brain (*N* = 5) or olfactory bulb (*N* = 5) genomic DNA was analyzed by NGS around the predicted Cas9 cleavage site of the *HTR2A* gene. We detected an indel frequency of 0.07–0.13% in all five brain samples (Fig. 3D). In all brain cases examined, the indel profile revealed single base pair deletions of adenine at identical positions near the PAM site (arrows, Fig. 3D). A recent report has indicated that the most abundant modification following CRISPR/Cas9 editing was 1 base pair deletion or insertion with 50% of these attributable to changes in adenine alone (33). Other indels observed were single base deletions in cytosine at the same position in three out of four brain cases and in guanine in one brain sample (Brain #4, Fig. 3D). A similar result was obtained in the olfactory bulb samples from the same five animals (Fig. S3). Interestingly, we observed more variety in the type of base pair deletions in olfactory bulb samples as compared with brain samples.

For the single base pair deletions in adenine (arrows Fig. 3D), these two indels would result in a predicted frameshift mutation leading to the insertion of premature stop codons (Fig. 3E). It is noteworthy that none of the indels revealed an insertion of the 200 bp donor SS ODN repair template, suggesting that NHEJ is the primary method of repair in generation of nonsense mutations. The low frequency of editing is not surprising given the large dilution effect of brain genomic DNA. Neurons make up less than 10% of the total number of cells in the brain, and we predict that only a subpopulation of these neurons would in fact be targeted by the AAV cargo. Taken together, these results support CRISPR-mediated gene editing of HTR2A in vivo that is most likely occurring through the single-nucleotide INDEL formation and generation of premature stop codons via NHEJ.

### Immunohistochemical analysis confirms HTR2A gene disruption in multiple CNS regions

**Fig. 4.**
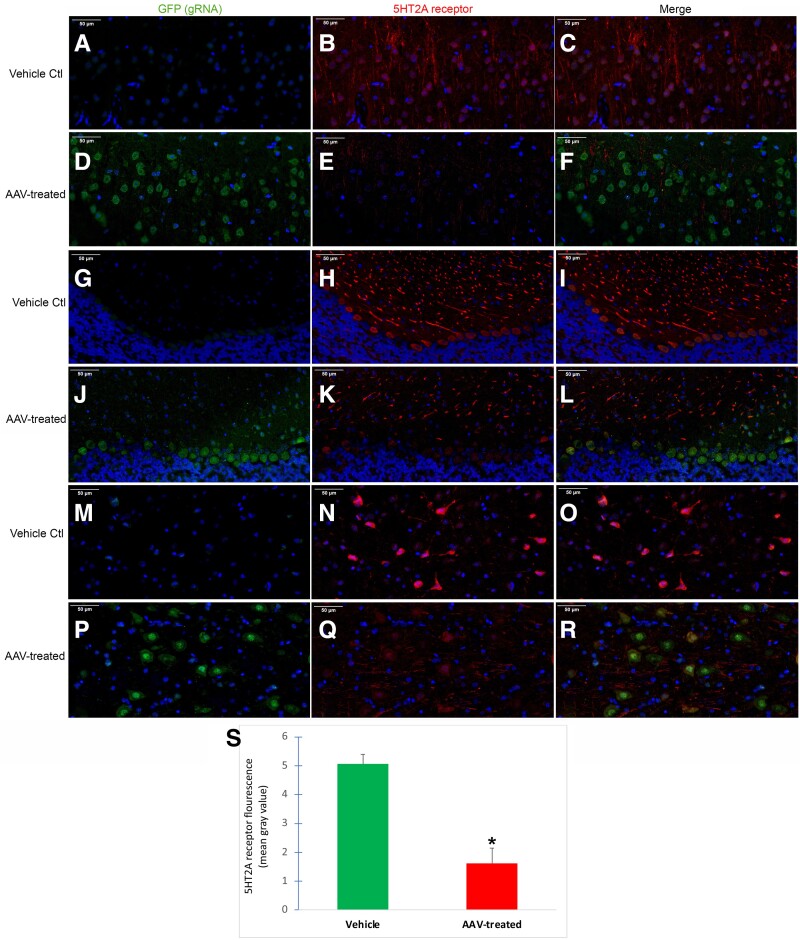
Intranasal AAV delivery of copackaged *Cas9* DNA and a *HTR2A*-targeting gRNA leads to a decrease in 5HT-2A receptor protein expression. Mice were treated with vehicle or AAV vectors on day 1, and 5 weeks later sacrificed, fixed, and 4-µm paraffin-embedded sagittal tissue sections were stained with anti-GFP (green, 1:1,000) or an antimouse 5HT-2A receptor antibody (red, 1:500). Whole slide imaging was performed using a Pannoramic Midi II scanner (see Methods for details). Representative 40× immunofluorescence images from vehicle controls or AAV-treated mice in three different brain regions: A–F) cortex, G–L), cerebellum, and M–R) pontine gray subcortical region. DAPI nuclear stain staining is indicated by blue. In all three brain regions, a general pattern emerged indicating less robust staining profile of the 5HT-2A receptor in cell body regions, as compared with apical dendrites extending away from the cell body. All scale bars represent 50 µm. Data are representative of *N* = 3 brains each for control and AAV-treated. S) Quantitative analysis using ImageJ software indicated a significant decrease in 5HT-2A receptor fluorescence intensity of AAV-treated mice (red bar) versus vehicle controls (green bar). Data represent the mean gray value ± SEM of three different brain regions (same as above) in each mouse (*N* = 3 for each group). Asterisk denotes significant difference, *P* = 0.0026.

Five weeks following intranasal AAV9 delivery of copackaged Cas9 DNA and a HTR2A-targeting gRNA, immunofluorescence studies were conducted in fixed, sagittal mouse sections to examine 5HT-2A receptor protein expression. gRNA expression was visualized by GFP fluorescence while 5HT-2A receptor labeling was accomplished utilizing an antibody previously demonstrated to be highly specific for the mouse 5HT-2A receptor (34). Colocalization of gRNA/GFP together with 5HT-2A receptor fluorescence was evident throughout the CNS including layers I–III of the cortex (Fig. 4D–F), the Purkinje cell layer of the cerebellum (Fig. 4J–L), and subcortical areas including within pontine gray nuclei (Fig. 4P–R). While strong 5HT-2A receptor labeling was evident in vehicle controls in these regions, in AAV-treated mice, staining appeared to be less robust (Fig. 4). As expected, there was little evidence of GFP (gRNA) staining in vehicle control mice. In AAV-treated mice, GFP (gRNA) labeling was strongest in the cell bodies of neurons, and when colocalized with 5HT-2A in AAV-treated mice, staining of 5HT-2A appeared weaker in the cell bodies with some labeling still apparent within apical dendrites. ImageJ quantification indicated a significant 68% decrease in 5HT-2A receptor fluorescence of AAV-treated mice as compared with vehicle controls (Fig. 4S), *P* = 0.0026. These results can be interpreted to suggest that treatment of the copackaged spCas9 DNA and a Htr2A-targeting gRNA led to a decrease in 5HT-2A receptor staining. Importantly, knockdown of the 5HT-2A occurred in brain regions linked to anxiety-mediated pathways including the IPN (31) and pontine gray nuclei (35).

### Intranasal AAV delivery of copackaged Cas9 DNA and a HTR2A-targeting gRNA decreases anxiety

Decades of research has supported the role of serotonin in stress-associated neurocircuitry and therapeutics such as the selective 5-HT uptake inhibitors (SSIRs) that down-regulate or other compounds that block serotonin receptors, as used in the treatment of anxiety disorders (36–41). The serotonin receptor most likely mediating anxiety is the 5HT-2A receptor, which is upregulated by various stressors (42). Conversely, anxiety is reduced in 5HT-2A receptor knockout mice (9, 10). We propose that the forebrain circuits underlying the potential anxiolytic effects of 5HT-2A receptor knockdown involve key nodal points in overlapping functions related to the generation and entrainment of circadian rhythms, the limbic cortices implicated in the etiology of anxiety, and the monoamine and thalamic nuclei interconnected with these limbic and circadian systems (43, 44). These ventral primarily limbic structures extend from the anterior hypothalamus to the midbrain and pons and include connections with the limbic cortices such as the orbital insular and hippocampal–amygdala cortices with rich connections to the epithalamic structures such as the medial and lateral habenula as well as the substantia nigra and ventral tegmental area, the raphe nuclei, and nearby locus coeruleus region (45). The locus coeruleus pathway includes areas implicated in stress including the habenula and the IPN (44).Therefore, we tested whether codelivery of Cas9 DNA and a Htr2a-targeting gRNA could decrease anxiety in mice using two well-characterized behavioral tests: marble burying (46) and light–dark box (47, 48).

**Fig. 5.**
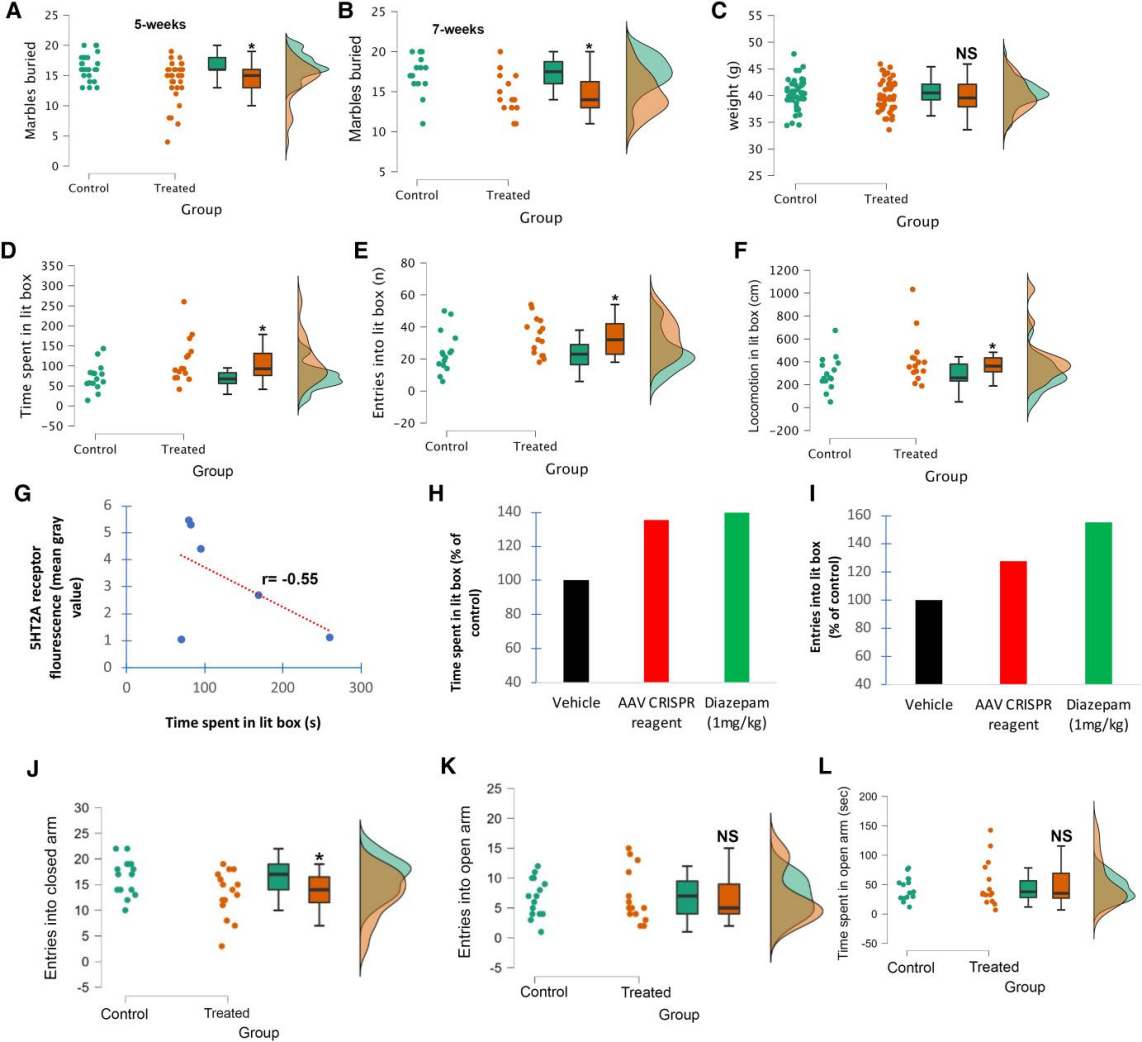
Intranasal AAV delivery of copackaged *Cas9* DNA and a *HTR2A*-targeting gRNA decreases anxiety. A, B) Mice were treated intranasally on day 1 with either 2.0 × 10^11^ A) or 4.0 × 10^11^ viral particles B) and compared with vehicle controls either 5 weeks A) or 7 weeks B) later in a marble burying paradigm. Results for 5-week AAV-treated mice indicated a 14.8% decrease in the number of marbles buried compared with vehicle controls (*N* = 30, *P* = 0.0008). Results were also significant at 7 weeks with AAV-treated mice showing a 13.2% decrease in the number of marbles buried (*N* = 15, *P* =0.013). C) There was no significant difference in weight in vehicle controls (40.4 ± 2.77, *N* = 45) and AAV-treated mice (40.0 ± 2.95, *N* = 45), *P* = 0.569. D–F) Mice were treated intranasally with vehicle or with 2.0 × 10^11^ viral particles on day 1, and 5 weeks later tested behaviorally using a light–dark paradigm. AAV-treated mice lead to a significant increase in the time spent in the lit box D), *P* = 0.025; the number of entries into the lit box E), *P* = 0.024; and total locomotion within the lit box F), *P* = 0.049. Data represent *N* = 15 animals in each group. For A–F), data represent the mean ± SEM, with G) correlation between the 5HT-2A receptor protein fluorescence following immunostaining with the time spent in lit box indicated a moderate negative relationship, *r* = −0.55. H, I) Comparison of AAV-treated mice at 5 weeks with acute treatment of diazepam (1 mg/kg p.o., 1 h) indicated comparable results in the time spent in lit box. Acute diazepam treatment led to a greater number of entries into the lit box area as compared with AAV-treated mice I). *N* = 15 for AAV-treated mice and *N* = 12 for diazepam-treated mice. J, K) Mice were treated intranasally on day 1 with either 2.0 × 10^11^ (green, *n* = 15) or vehicle (orange, *n* = 15) and tested 5 weeks later in an EPM paradigm. Following AAV treatment, there was a significant decrease in the number of entries in the closed arms J), *P* = 0.017. K) There was no significant difference in the number of entries into the open arm between the two groups (*P* = 0.50). L) A 17% increase in the time spent in the open arm was documented; however, results did not reach significance (*P* = 0.257). Asterisk denotes statistical significance between the two groups (*P* < 0.05). NS denotes nonsignificant (*P* > 0.05). Data represent the average ± SEM.

The marble burying test is used to record the number of marbles buried by mice placed in a novel environment. The theoretical basis for marble burying as an anxiety-related behavior stems from findings that it is reduced by anxiolytics/SSRIs and from the well-documented innate response of mice to bury threating objects (49, 50). Results for 5-week AAV-treated mice indicated a 14.8% decrease in the number of marbles buried compared with vehicle controls (*N* = 30, *P* = 0.0008) (Fig. 5A). Similar results were obtained following a doubling of the dose at 7 weeks post treatment (*N* = 15, *P* = 0.013) (Fig. 5B). Following treatment of mice with copackaged Cas9 DNA and gRNA on day 1, there were no reported observational differences in behavior of mice and treated mice did not differ significantly in weight at the end of the 5-week trial (*P* = 0.569) (Fig. 5C), suggesting there were no major off-target edits in critical genes.

The light/dark box test is also a well-characterized method for evaluating the relative anxiety status of mice (47). The light/dark paradigm in mice is based on a conflict between the innate aversion to brightly illuminated areas and the spontaneous exploratory activity. If given a choice, mice prefer the dark box and anxiolytic compounds have been found to increase the total duration of time spent in the lit area as well as the number of entries into the lit box area (48). AAV-treated mice led to a significant increase compared with vehicle controls in the time spent in the lit box (35.7% increase, *P* = 0.024), the number of entries into the lit box (27.5% increase, *P* = 0.024), and total locomotion within the lit box (27.8%, *P* = 0.049) (Fig. 5D–F). A regression analysis between total 5HT-2A receptor protein fluorescence and time spent in the lit box indicated a moderate negative linear correlation (*r* = −0.55) (Fig. 5G). For context, we compared these results with the acute effects of diazepam (1 mg/kg p.o., 1 h). As shown in Fig. 5, AAV treatment led to comparative results to diazepam in the time spent in the lit box (35.7% versus 40%) (Fig. 5H), with diazepam outperforming AAV-treated mice in the number of entries into the lit box (27.5% versus 55%) (Fig. 5I). Thus, intranasal AAV delivery of copackaged Cas9 DNA and a Htr2a-targeting gRNA significantly decreased anxiety and performed on par with the benzodiazepine, diazepam.

We also tested the ability of the designed CRISPR/Cas9 cargo to decrease anxiety utilizing the EPM test, a common test used to evaluate anxiety in rodents (51). In this case, a different species of mice was tested (C57Bl/6J); otherwise, identical treating protocols were employed. At 5 weeks postdosing, mice exhibited a significant 24% decrease in the number of entries into the closed arm (Fig. 5J) (*P* = 0.017). In addition, the AAV-treated cohort resulted in less distance traveled into closed arms (*P* = 0.032). There was no significant difference between the two groups in the number of entries into the open arm (*P* = 0.50) (Fig. 5K). A documented 17% increase in the time spent in open arms was observed; however, this did not reach statistical significance due to the large variance observed in the AAV-treated group (*P* = 0.257) (Fig. 5L). There were no significant differences in weight between the two groups (*P* = 0.183).

Finally, to test whether the designed CRISPR/Cas9 cargodecreased depressive-like behavior, tail suspension tests were performed. The tail suspension test in rodents is a commonly used behavioral test that is often used to assess depression-like behavior in animal models (52). This test is based on the principle that when mice or rats are suspended by their tails, they will initially struggle to escape but eventually become immobile. The duration of immobility is thought to reflect the animal's level of helplessness and is often used as a measure of depression-like behavior. Tail suspension experiments were undertaken 5 weeks post AAV9–CRISPR/Cas9 treatment in mice and the results are depicted in Fig. S4. As shown, there was no significant difference in the total immobility time between vehicle controls and AAV9-treated groups (*P* > 0.05). These results suggest that decreasing 5HT-2A receptor density does not promote antidepressive actions in mice. However, it is important to point out that while the tail suspension test has been shown to be sensitive to a variety of antidepressant treatments in rodents, it is important to use caution when interpreting the results (53). There have been some concerns surrounding the use of the tail suspension test as a measure of depression-like behavior in mice including its reliability and validity as a measure of depression. In addition, studies have reported that the tail suspension test may be influenced by factors other than depression, such as motor impairment (54).

## Conclusions

The World Health Organization reports that anxiety disorders affect 264 million people worldwide, with a 25% rise since the beginning of the Covid-19 pandemic (55). Only 60–85% of patients with anxiety disorders respond to current therapeutic treatments (56). Patients with treatment-resistant anxiety represent a significant ongoing challenge and underscore the necessity for novel treatment methodologies. Targeting the down-regulation of 5HT-2A receptors constitutes a rational approach based on the mechanism of action of current medications (7, 9). In the present study, we show that delivery of copackaged Cas9 DNA with a Htr2a-targeting gRNA led to a sharp increase in indel formation and decreased neuronal electrical activity in vitro without any observable toxicity. In vivo, AAV9 treatment of mice generated single base pair deletions leading to the introduction of premature stop codons as well as a concomitant down-regulation of the 5HT-2A receptor. The subsequent decreased expression of functional 5HT-2A receptor reduced anxiety-related behavior in two different species of mice using three different behavioral paradigms. Our results suggest that even with a low percentage of neuronal gene editing, significant anxiolytic effects are observed. Future directions to improve efficacy should be focused on improving Cas9 cutting efficiency that relies on NHEJ and not HDR systems to introduce donor sequence templates. In addition, improved efficacy could be obtained by increasing the directional flow of cargo following intranasal delivery. Techniques that could be applied in this manner include pulsed ultrasound and transcranial magnetic stimulation (57, 58). Delivery within the CNS was accomplished using a noninvasive intranasal delivery platform that can bypass the BBB, typically a major hurdle for large cargo such as CRISPR/Cas9. Our delivery platform of CRISPR/Cas9 and proof-of-concept results indicate that certain traits can be directly modified long-term, and that this has important implications for the next generation of psychotropic medications. Specifically, precision targeting of the 5HT-2A receptor by CRISPR/Cas9 gene editing may provide a new direction for patients who exhibit treatment-resistant anxiety or depression (13, 14).

## Supplementary Material

pgad170_Supplementary_Data

## Data Availability

The authors confirm that the data supporting the findings of this study are available within the article and/or its supplementary materials.
