# Supplementary material for: Genetic modulation of the HTR2A gene reduces anxiety-related behavior in mice
Source: PNAS Nexus. 2023 Jun 20;2(6):pgad170. doi: 10.1093/pnasnexus/pgad170 (PMC10281383; doi:10.1093/pnasnexus/pgad170)

**Figure S1. Experimental approach to validate HTR2A gene knockdown**. Overview of flow-through to generate founder mice. Step 1: Gene modification in mouse ESCs using designed CRISPR/spCas9, gRNA sequence ***TGCAATTAGGTGACGACTCGAGG*,** and a 200 by donor sequence containing two stop codons. Step 2: Confirmed ESC cells harboring KI insert are expanded (cloned). Step 3: Following expansion, 3-5 ESCs were injected into each mouse blastocysts. Step 4: Embryos are then transferred to surrogate female mice and resulting founder F_0_, chimeric mice were analyzed for KI insert by PCR and RFLP.


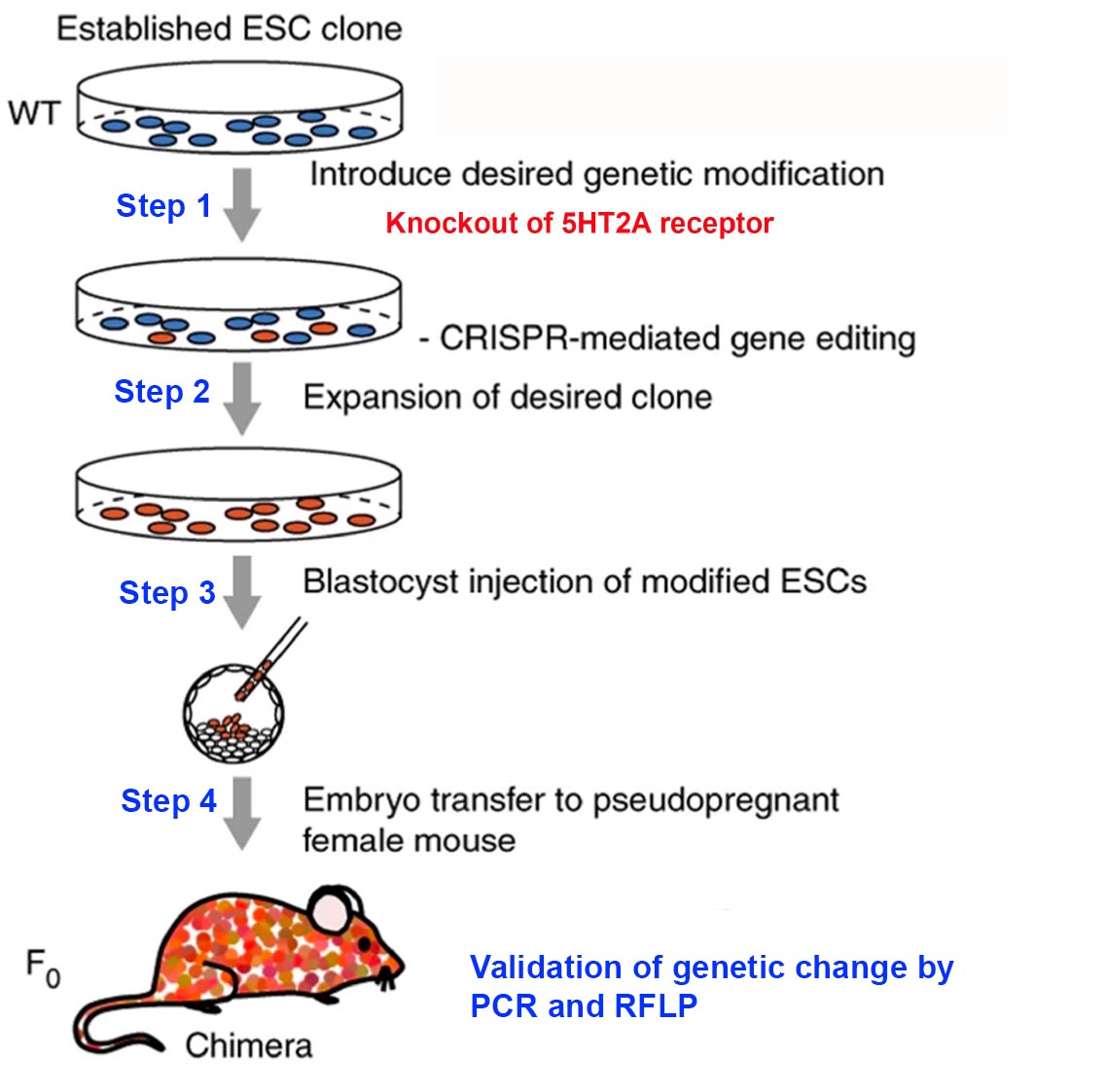


Modified from Huijbers et al., Nat Protoc 10, 1755-1785 (2015)

**Figure S2. Validation of CRISPR/Cas9 strategy in knock-in mice for the *HTR2A* receptor**. The *HTR2A* gene encodes a single protein-coding transcript, *Htr2a*-201. The strategy involved inserting a STOP-pA cassette at the start of all protein coding transcripts. Gene editing was carried out in mouse ESC cells (C57BI/5N background) following transfection of ESC cells with Cas9 plasmid, gRNA and STOP-pA cassette donor sequence as described in Figure 1 (main text). Following validation by sequencing, blastocysts were injected with positive ESC clones and subsequent embryos were implanted in surrogate mice. Data show the results of 18 founder mice. Full-length *HTR2A* genotyping by PCR of tail DNA indicated positive mice for the mutant band in animals 7-10. Further verification in animals 7-10 was confirmed by both Sanger sequencing (A) and RFLP (B)

1. Htr2a full length Genotyping PCR

Expected Products:

- - WT: 375 bps
  - Mutant: 464 bps


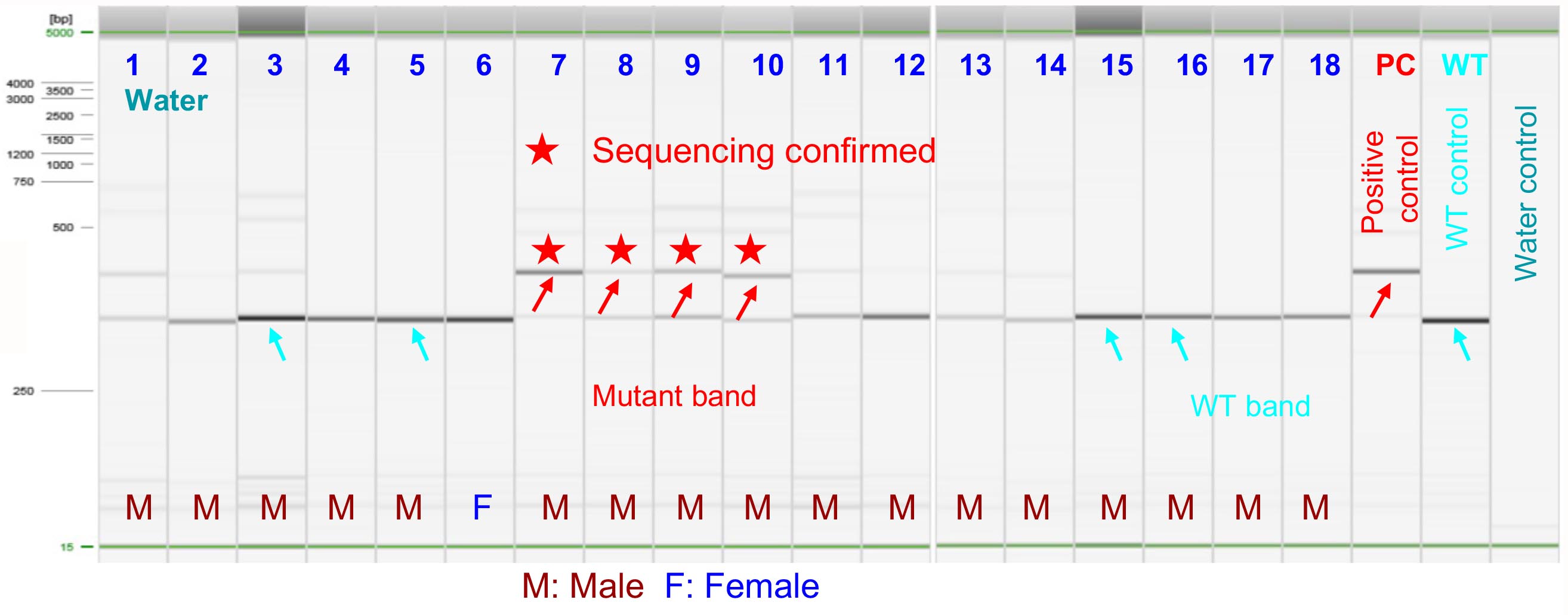


**B.** Htr2a Genotyping PCR RFLP

- Enzyme:
  - EcoRI
- Expected Products:
  - WT: 375 bps (No cuts)
  - Mutant: 198 + 266 bps


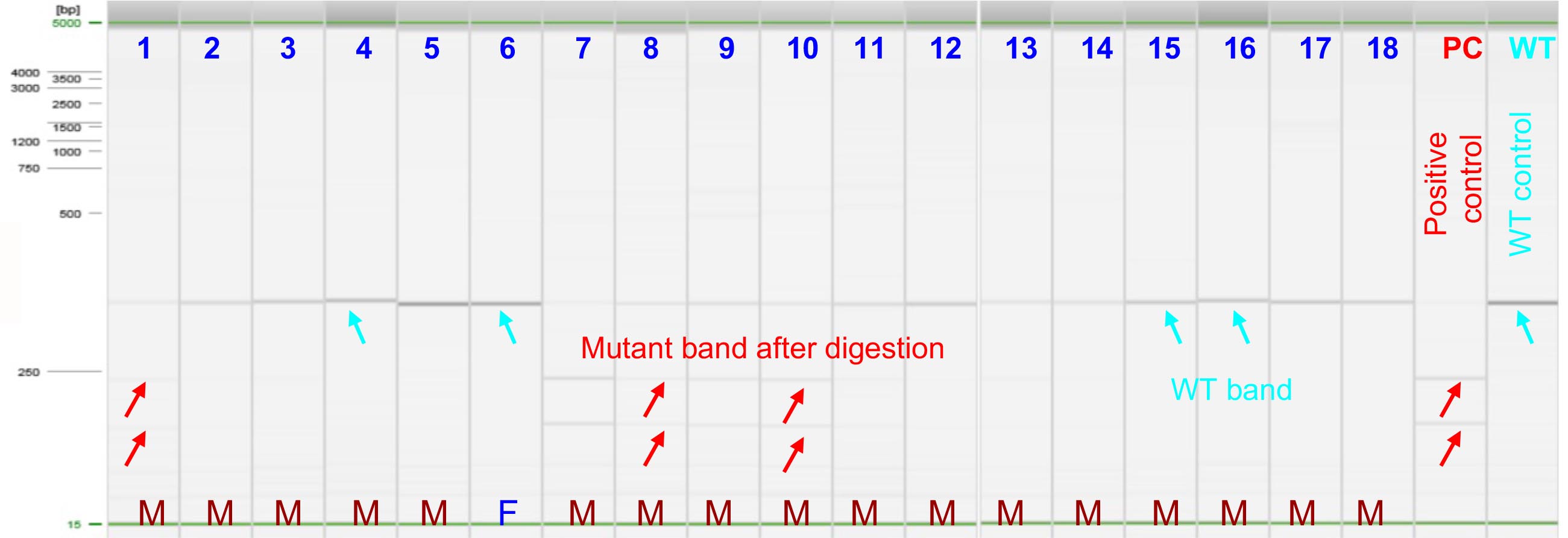


**Figure S3:** Next-generation sequencing analysis on-target effects in olfactory bulb samples from five separate mice were analyzed and the most prevalent reads obtained are shown. The profile mutations induced by *HTR2A*-targeting AAV-CRISPR/Cas9 were all single base-pair deletions indicated by the -1 symbol. PAM, protospacer adjacent motif; WT, wild type.


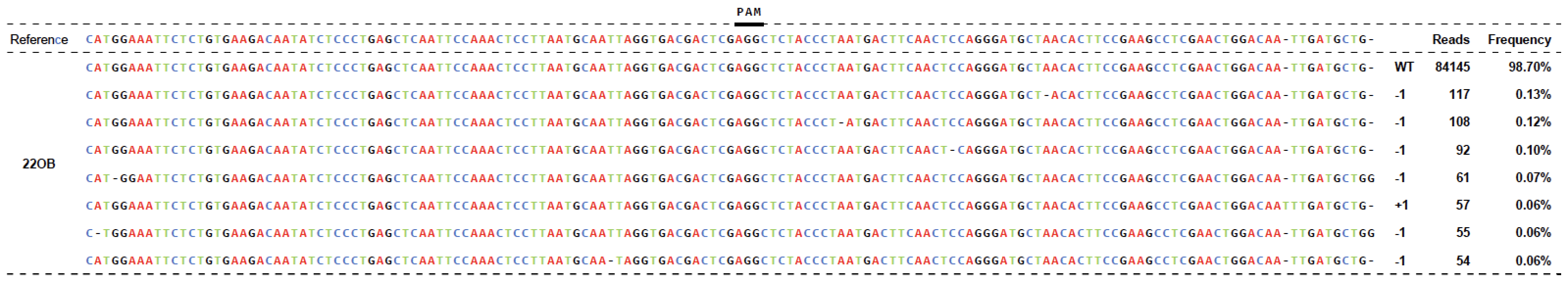


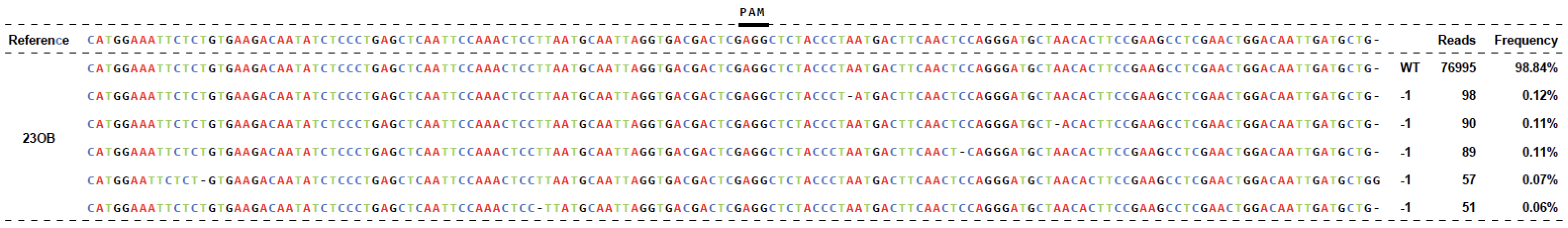


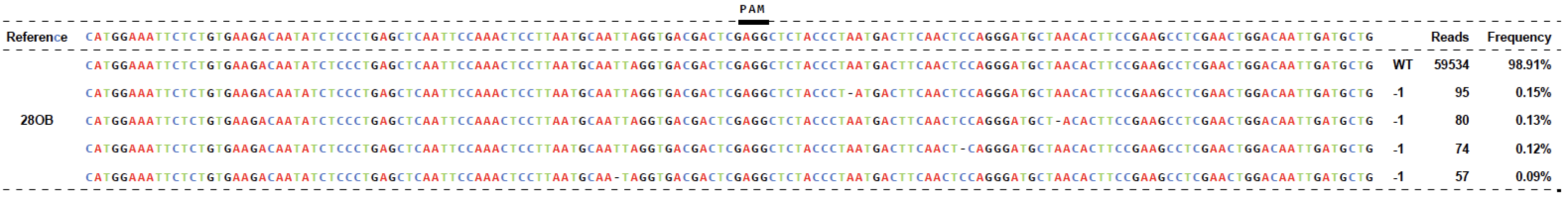


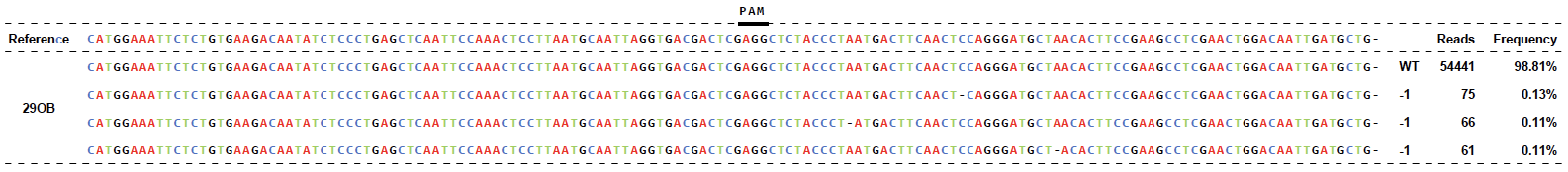


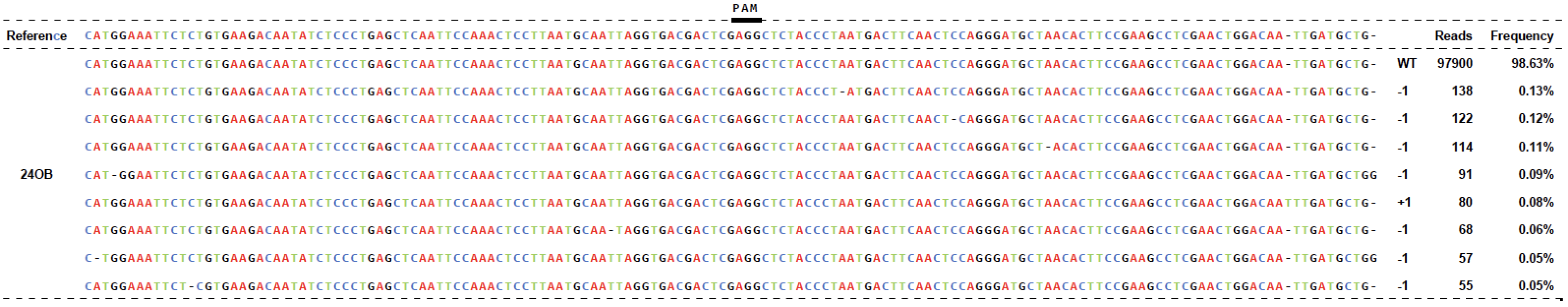


**Figure S4:** **Intranasal adeno-associated virus delivery of co-packaged *Cas9* DNA and a *HTR2A*-targeting gRNA has no effect in a depression-like paradigm.** Mice were treated intranasally on day 1 with either 2.0 x 10^11^ (green, n=15) or vehicle (orange, n=15) and 5 weeks later tested using a tail suspension behavioral paradigm (See methods for details). In this test, mice will tend to actively struggle to right themselves when suspended upside down by the tail. The tail suspension test is an indicator of depression and measures the amount of time the mouse struggles to right itself before “giving up” and becoming immobile. A short time to immobility indicates depression. The results indicated there was no significant difference between the two groups (p-value >0.05).


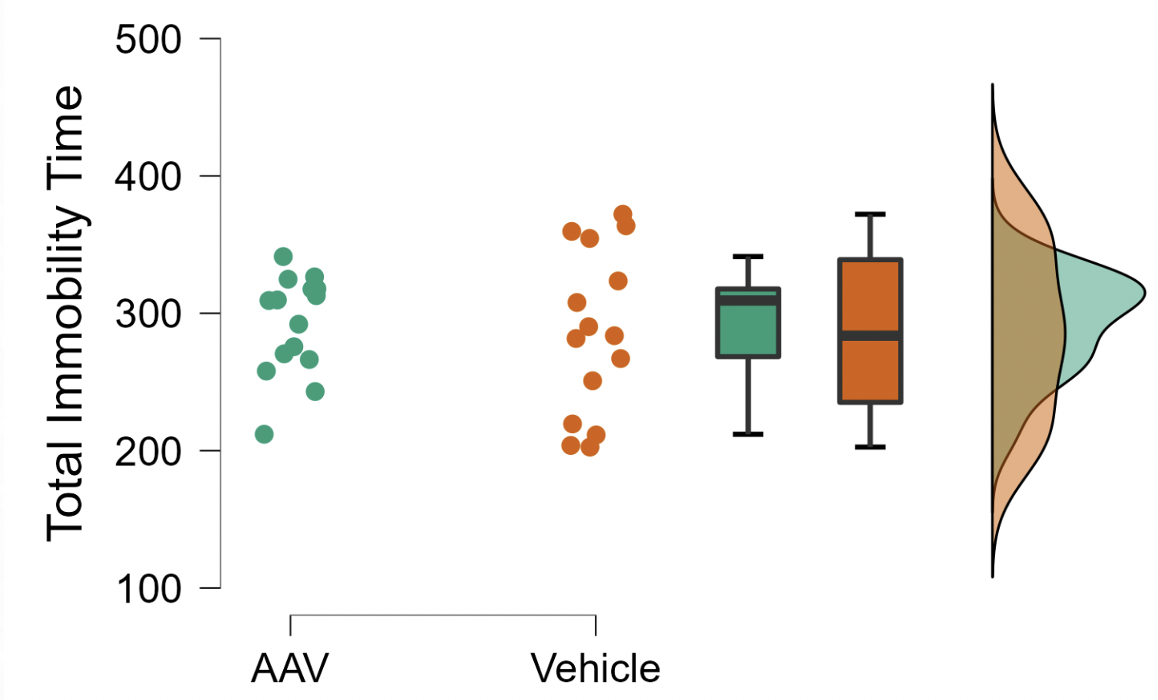

Supplement: pgad170_Supplementary_Data [file pgad170_supplementary_data.docx]
